# Metagenomic and metabolomic profiling in primary aldosteronism with coexisting obstructive sleep apnea

**DOI:** 10.3389/fendo.2026.1858100

**Published:** 2026-07-20

**Authors:** Li Yang, Yi Tao, Yan He, Shijie Liu, Lulu Gan, Anni Dai, Qing Ni, Yun Wang, Fengping Li, Qian Liu, Yang Hu, Yanqi Wang, Wugan Lu

**Affiliations:** 1 Yan’an Hospital Affiliated to Kunming Medical University, Kunming Technical Diagnosis and Treatment Center for Refractory Hypertension, Kunming, China; 2 Kunming Technical Diagnosis and Treatment Center for Refractory Hypertension, Kunming, Yunnan, China; 3 Department of Respiratory and Critical Care Medicine, Pu’er People’s Hospital, Pu’er, Yunnan, China; 4 Geriatrics, Liangshan Yi Autonomous Prefecture Hospital of Integrated Traditional Chinese and Western Medicine, Liangshan, Sichuan, China; 5 Electrophysiology Room in Cardiovascular Medicine Department, People’s Hospital of Yuxi City, Yuxi, Yunnan, China

**Keywords:** fecal metabolomics, gut microbiome, obstructive sleep apnea, primary aldosteronism, shotgun metagenomics

## Abstract

**Background:**

Primary aldosteronism (PA) frequently coexists with obstructive sleep apnea (OSA), and this comorbidity is associated with increased cardiometabolic risk. Although both PA and OSA have been individually linked to gut microbiome alterations, it remains unclear which layer of gut microbiome-associated variation best reflects clinical heterogeneity in PA with coexisting OSA.

**Methods:**

In this prospective observational study, we performed shotgun metagenomic sequencing and untargeted fecal metabolomic profiling in 29 adults with clinically confirmed PA, who were stratified according to OSA severity (G1-G4) based on overnight polysomnography. Microbial gene richness, taxonomic composition, functional potential based on KEGG annotation, and antibiotic resistance gene profiles were analyzed using standardized bioinformatic workflows. Metabolomic variation was assessed using multivariate analysis, pathway enrichment, and additional exploratory analyses incorporating apnea-hypopnea index (AHI) as a continuous variable. Multiple-testing correction was applied to metabolite-level comparisons.

**Results:**

Global gut microbial gene richness, alpha diversity, beta diversity, and broad functional profiles did not show strong group-level separation across OSA severity strata. Additional analyses using AHI as a continuous variable similarly showed no significant association between AHI and overall gene richness or alpha diversity indices. Nevertheless, selective genera showed exploratory associations with AHI, suggesting that localized taxonomic signals may occur despite relative stability of global community structure. Antibiotic resistance gene profiles showed marked inter-individual variability without clear group-level separation, although ARO richness showed an exploratory inverse association with AHI. In contrast, fecal metabolomic profiling revealed nominal phenotype-associated differences, including trehalose-related metabolites and FAHFA species that showed inverse exploratory associations with AHI. However, no individual metabolite remained significant after global Benjamini–Hochberg false discovery rate correction.

**Conclusions:**

In PA with coexisting OSA, gut microbiome-associated heterogeneity appears to be more readily reflected by selected taxonomic and metabolic signals than by global microbial diversity or broad functional potential. However, given the small sample size, limited control of clinical and lifestyle confounders, and lack of metabolite-level significance after global FDR correction, these findings should be interpreted as exploratory and hypothesis-generating. Larger controlled cohorts incorporating PA subtype, medication exposure, dietary assessment, and longitudinal validation are needed.

## 1 Introduction

Primary aldosteronism (PA) is a common and underdiagnosed cause of secondary hypertension and is increasingly recognized as a major driver of cardiovascular risk beyond blood pressure elevation alone. Contemporary evidence indicates that PA is present in a meaningful fraction of patients with hypertension in primary care, with the prevalence increasing alongside hypertension severity, supporting more systematic screening in high risk populations (1, 2). More recently, the 2025 Endocrine Society Clinical Practice Guideline further emphasized broader case detection by suggesting that all individuals with hypertension be screened for PA using aldosterone, renin, and the aldosterone-to-renin ratio (3). Importantly, patients with PA experience higher rates of cardiovascular events compared with those with essential hypertension, even after accounting for conventional risk factors, highlighting aldosterone excess as a disease mechanism rather than a mere biomarker (4). In parallel, obstructive sleep apnea (OSA) is highly prevalent in adults and is strongly linked to hypertension, cardiometabolic dysregulation, and adverse cardiovascular outcomes through intermittent hypoxia, sympathetic activation, and systemic inflammation (5, 6).

Accumulating clinical data suggests that PA and OSA frequently coexist, particularly in patients with difficult to control hypertension, and that the relationship may be bidirectional. In resistant hypertension, aldosterone status has been associated with OSA severity, supporting a mechanistic link between mineralocorticoid excess and sleep disordered breathing (7). Population level and clinical studies further indicate that fluid retention and rostral fluid shift may contribute to upper airway collapsibility, providing a plausible pathway by which aldosterone excess can aggravate OSA, while OSA related intermittent hypoxia and neurohumoral activation may also influence the renin-angiotensin-aldosterone system (8, 9). Despite these observations, the biological basis of this comorbidity remains incompletely understood, and currently available explanations do not fully account for the heterogeneity in clinical presentation and metabolic risk among patients with PA and coexisting OSA.

The gut microbiome has emerged as a key regulator of host metabolism and immune inflammatory signaling, offering a systems level framework to connect endocrine and cardiometabolic phenotypes with environmental and lifestyle exposures (10). In hypertension research, converging evidence supports links between microbial community features, microbial metabolites, and blood pressure regulation, including pathways involving short chain fatty acids and other microbe derived bioactive compounds (11, 12). OSA is also increasingly associated with gut microbiome alteration. Experimental models demonstrate that intermittent hypoxia can reshape gut microbial communities (13), and human studies report associations between hypoxia burden and gut microbiota composition, microbial functions, and circulating metabolites, suggesting that microbiome linked metabolic outputs may represent a sensitive readout of OSA related physiological stress (14, 15).

Evidence linking the gut microbiome to aldosterone related phenotypes is emerging but remains limited, particularly in clinically defined PA. Recent work has reported gut microbiota alterations in patients with PA and suggested relationships with metabolic disorders (16), while broader perspectives propose interactions between the gut microbiome and the renin-angiotensin-aldosterone system through immune mediated pathways (17). In addition, the gut ecosystem constitutes a large reservoir of antibiotic resistance genes (ARGs), and resistome features can vary across populations and exposures, potentially reflecting broader ecological perturbations relevant to cardiometabolic health (18, 19). However, studies that jointly profile taxonomic composition, functional potential, metabolomic outputs, and resistome patterns in a stratified PA plus OSA context remain scarce.

Therefore, we asked whether gut microbiome-associated heterogeneity in PA with coexisting OSA is better reflected in microbial community structure, genetic functional potential, resistome features, or metabolic outputs. We hypothesized that, in this comorbidity, the gut ecosystem may retain a relatively stable global architecture while exhibiting selective shifts in specific taxa and downstream metabolic readouts. To address this question, we performed shotgun metagenomic sequencing and untargeted fecal metabolomics in clinically stratified PA patients with OSA, profiling taxonomic composition, KEGG functional annotations, ARG patterns, and metabolomic variation. By examining these layers together and incorporating additional exploratory analyses using apnea-hypopnea index as a continuous measure of OSA severity, this study aimed to assess which microbiome-associated readouts may best capture clinical heterogeneity in PA with coexisting OSA.

## 2 Methods

### 2.1 Study design and participants

A prospective observational study was conducted involving 29 adult patients (≥18 years) diagnosed with primary aldosteronism (PA) at the Hypertension Center of Yan’an Hospital, Kunming, China, between January and December 2023. The study protocol was approved by the Medical Science Research Ethics Committee of Yan’an Hospital (2018-073-02), and written informed consent was obtained from all participants prior to enrollment.

The diagnosis of PA followed a two-step procedure, including initial screening based on an elevated aldosterone–renin ratio (ARR > 30) and confirmatory testing using the saline infusion test. The saline infusion test was performed in the recumbent position, and PA was confirmed based on a post-infusion plasma aldosterone concentration > 10 ng/dL. All enrolled patients underwent overnight polysomnography for evaluation of sleep-disordered breathing. Obstructive sleep apnea (OSA) was diagnosed based on an apnea–hypopnea index (AHI) ≥ 5 events per hour, calculated as the average number of apnea and hypopnea events per hour of sleep. OSA severity was classified as mild (5 ≤ AHI < 15), moderate (15 ≤ AHI < 30), or severe (AHI ≥ 30).

Exclusion criteria included gastrointestinal diseases or prior intestinal surgery, use of antibiotics, probiotics, or other microbiota-altering medications within one month prior to fecal sampling, sleep disorders other than OSA, secondary causes of hypertension unrelated to OSA, severe hepatic or renal dysfunction, active infection, recent acute cardiovascular or cerebrovascular events, and malignant neoplasms.

### 2.2 Demographic and clinical data collection

Demographic characteristics, including age, sex, height, weight, and body mass index (BMI), as well as medical history of diabetes mellitus and coronary artery disease, were obtained from the hospital information system. Fasting venous blood samples collected at 07:00 were analyzed for ARR, serum potassium, fasting glucose, total cholesterol, triglycerides, high-density lipoprotein cholesterol, and low-density lipoprotein cholesterol using standard clinical laboratory methods.

Overnight polysomnography was performed using the SOMNOtouch RESP^®^ system, with continuous monitoring for a minimum of 7 hours. Ambulatory blood pressure monitoring was conducted using an Oscar 2 device (SunTech Medical, Inc.), with measurements recorded at 30-minute intervals during daytime (06:00–22:00) and 60-minute intervals during nighttime (22:00–06:00).

### 2.3 Shotgun metagenomic sequencing and bioinformatic processing

Fresh fecal samples were aseptically collected, immediately snap-frozen in liquid nitrogen, and stored at −80 °C until analysis. All samples were transported on dry ice to Guangzhou KingMed Diagnostics Group Co., Ltd. for shotgun metagenomic sequencing and downstream analyses. Microbial DNA was extracted using the QIAamp Fast DNA Stool Mini Kit (Qiagen, Hilden, Germany) according to the manufacturer’s instructions. Sequencing libraries were prepared using the NEXTFLEX Rapid DNA-Seq Kit (Bioo Scientific, Austin, TX, USA) and sequenced on the Illumina NovaSeq platform to generate paired-end 150-bp reads.

Raw reads were quality filtered using fastp (v0.23.2) (20), including adapter trimming, removal of reads containing more than 10% ambiguous bases, and trimming of low-quality bases with Phred scores < 20. Host-derived reads were removed by aligning filtered reads to the human reference genome (GRCh38) using Bowtie2 (v2.4.5) (21), retaining only unmapped reads for subsequent analyses. *De novo* assembly was performed using MEGAHIT (v1.2.9) with k-mer sizes ranging from 21 to 121 (22, 23). Assembled contigs were fragmented at ambiguous bases to generate scaftigs, and sequences shorter than 500 bp were excluded (24, 25).

Open reading frames were predicted from assembled scaftigs using MetaGeneMark (v3.38) (26–30). A non-redundant gene catalog was constructed using CD-HIT (v4.8.1) with a sequence identity threshold of 95% and coverage of 90% (31, 32). Clean reads from each sample were mapped back to the non-redundant gene catalog using Bowtie2 for gene abundance estimation, and genes with read counts greater than two in at least one sample were retained. Taxonomic annotation was performed by aligning predicted genes to the NCBI non-redundant protein database (December 2023 release) using DIAMOND (v2.1.6) (33) in blastx mode with an e-value threshold of 1e−5. Taxonomic assignments were determined using the lowest common ancestor algorithm implemented in MEGAN6 (v6.24.1), and gene abundances were aggregated at different taxonomic levels for downstream analyses.

### 2.4 Untargeted metabolomic profiling and data processing

Fecal samples (100 mg) were homogenized in liquid nitrogen and extracted with 80% methanol. After vortexing and incubation on ice, samples were centrifuged at 15, 000 × g for 20 minutes at 4 °C. The supernatant was diluted with MS-grade water to achieve a final methanol concentration of 53% and centrifuged again under identical conditions. The resulting supernatants were subjected to ultrahigh-performance liquid chromatography–mass spectrometry (UHPLC–MS) analysis. Pooled quality control (QC) samples were prepared by combining equal aliquots of representative samples and processed in parallel with study samples.

Metabolite separation was performed using a Vanquish UHPLC system (Thermo Fisher Scientific) equipped with a Hypersil Gold C18 column (100 mm × 2.1 mm, 1.9 μm) maintained at 40 °C. The mobile phases consisted of 0.1% formic acid in water (A) and methanol (B), delivered at a flow rate of 0.2 mL min^-1^ using a predefined gradient. Mass spectrometric detection was carried out in both positive and negative electrospray ionization modes, with full-scan acquisition over an m/z range of 100–1500 and data-dependent MS/MS acquisition.

Raw MS data were processed using Compound Discoverer software (version 3.3, Thermo Fisher Scientific). Feature extraction and alignment were performed with a mass tolerance of 5 ppm, followed by peak area normalization using QC samples. Metabolic features with coefficients of variation greater than 30% across QC replicates were excluded. Metabolite identification was achieved through accurate mass matching and MS/MS spectral comparison against mzCloud, mzVault, and an in-house database. Identified metabolites were mapped to Kyoto Encyclopedia of Genes and Genomes (KEGG) pathways for downstream enrichment analysis.

### 2.5 Statistical analysis

Statistical analyses of clinical variables were performed using SPSS Statistics version 22.0 (IBM Corp.) and R software (version 4.5.1) (34). Categorical variables were expressed as counts and percentages and compared using the chi-square test or Fisher’s exact test. Continuous variables were presented as mean ± standard deviation for normally distributed data or median with interquartile range for non-normally distributed data and compared using one-way analysis of variance or Kruskal–Wallis test, as appropriate.

Microbial alpha diversity was assessed using Shannon, Chao1, observed species, and Simpson indices. Beta diversity was evaluated based on Bray–Curtis dissimilarity and visualized using principal coordinate analysis. Permutational multivariate analysis of variance (PERMANOVA) was used to assess differences in community composition and R^2^ values were reported as effect size estimates. For alpha diversity and gene richness comparisons across OSA severity groups, Kruskal–Wallis tests were supplemented with epsilon-squared effect sizes. For comparisons between the two extreme groups, Cliff’s delta was calculated as an additional non-parametric effect size estimate.

To address OSA severity as a continuous clinical variable, exploratory Spearman correlation analyses were performed between AHI and selected microbiome-associated readouts, including gene richness, alpha diversity indices, representative taxa identified by LEfSe, antibiotic resistance gene metrics, and representative metabolites. Mean oxygen saturation was also examined in exploratory correlation analyses when relevant.

Differential microbial taxa between extreme phenotypes were identified using linear discriminant analysis effect size (LEfSe), with taxa exhibiting LDA scores greater than 2.0 considered discriminative. Antibiotic resistance gene profiles were analyzed based on Comprehensive Antibiotic Resistance Database annotations at the Antibiotic Resistance Ontology (ARO) level. Total ARG relative abundance, ARO richness, ARO Shannon diversity, ARO-level Bray–Curtis PERMANOVA, and pairwise ANOSIM results were summarized. Available CARD resistance mechanism annotations were also summarized to further characterize resistome features.

Metabolomic data were analyzed using principal component analysis and partial least squares–discriminant analysis, with PLS-DA used for descriptive visualization only. Differential metabolites were initially defined based on variable importance in projection scores greater than 1.0 combined with univariate statistical testing. In response to multiple-testing concerns, Benjamini–Hochberg false discovery rate correction was additionally applied across all detected metabolites within each ion mode. Metabolite-level findings were therefore interpreted as exploratory when they did not remain significant after global FDR correction. For differential taxonomic and metabolomic analyses, comparisons were primarily performed between the two extreme groups (G1 and G4) to enhance sensitivity, while AHI-based analyses were used as complementary exploratory analyses.

## 3 Results

### 3.1 Gene richness and shared gene profiles of the gut microbiome

**Figure 1:**
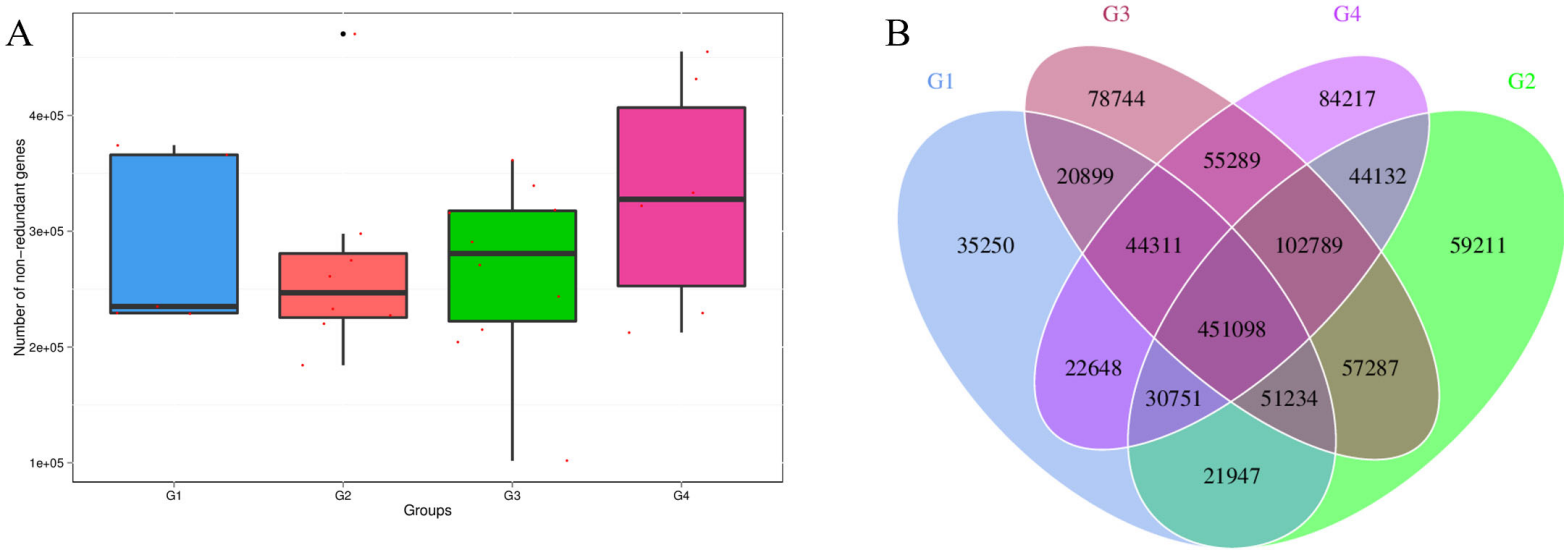
Gene richness and shared gene profiles across the four groups. **(A)** Comparison of non-redundant gene counts (gene richness) across groups G1 to G4. Each point represents an individual sample, and boxes indicate the interquartile range. **(B)** Venn diagram showing unique and shared genes among the four groups, including 451, 098 genes common to all groups. The Venn diagram is used to visualize gene set overlap and does not imply statistical inference.

To characterize the overall gene content of the gut microbiome across OSA severity groups, we first compared non-redundant gene richness and shared gene profiles among G1 to G4. As shown in **Figure 1A**, per sample non-redundant gene richness exhibited comparable distributions across the four groups, with no marked differences in overall gene counts. Consistently, Kruskal–Wallis analysis showed no significant difference in gene richness among groups (H = 1.818, *p* = 0.611), with a negligible effect size (epsilon-squared ≈ 0; **Supplementary Table 7**). Comparison between the two extreme groups also showed only a small effect size (G1 vs. G4 Cliff’s delta = -0.200, *p* = 0.662; **Supplementary Table 7**).

To further address OSA severity as a continuous clinical variable, we examined the association between apnea–hypopnea index (AHI) and gene richness. Spearman correlation analysis showed no significant association between AHI and non-redundant gene number (rho = 0.105, *p* = 0.589; **Supplementary Table 6**), indicating that increasing OSA severity was not accompanied by a monotonic change in global microbial gene richness in this cohort.

To examine the overlap of microbial gene repertoires, a Venn diagram was constructed based on non-redundant gene catalogs from each group (**Figure 1B**). A substantial proportion of genes (451, 098) was shared across all four groups, indicating a high degree of commonality in gut microbial gene content. Together, these results suggest that the global gene repertoire of the gut microbiome was broadly conserved across OSA severity strata in PA patients, although this finding should be interpreted in the context of the limited sample size.

### 3.2 Microbial diversity analysis of gut microbiota

**Figure 2:**
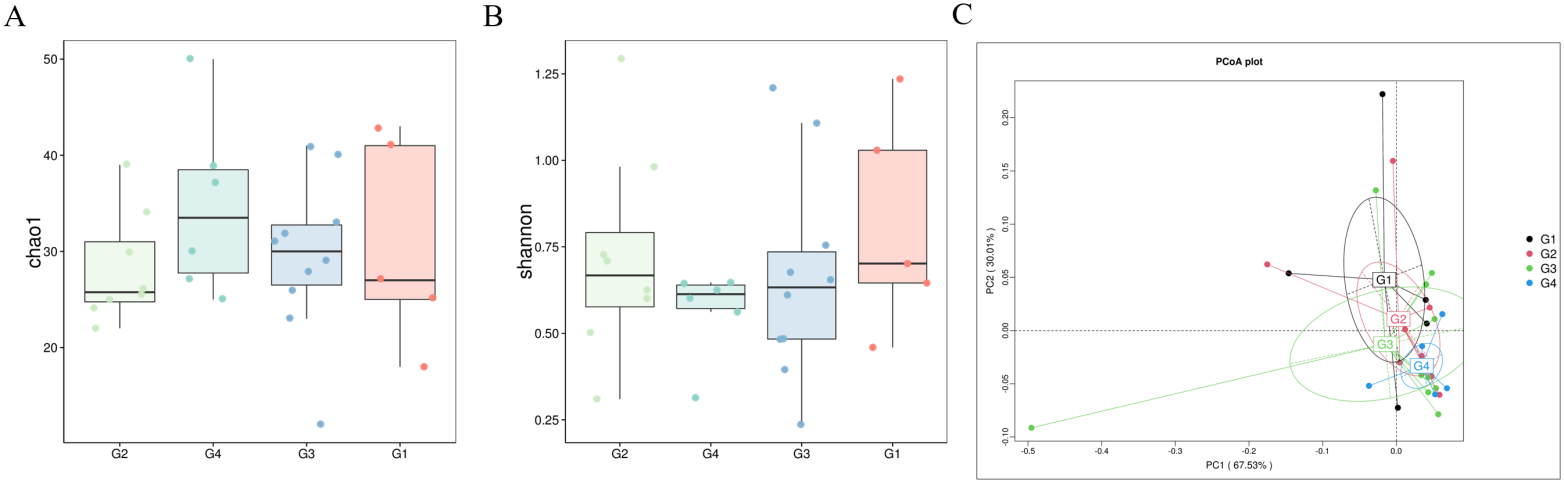
Diversity analysis of gut microbiota among the four groups (G1–G4). Alpha diversity was evaluated using Chao1 richness **(A)** and Shannon diversity index **(B)**. beta diversity was assessed using Principal coordinates analysis (PCoA) based on Bray–Curtis dissimilarity **(C)**. PERMANOVA showed that OSA severity groups explained only a small proportion of taxonomic variation at the genus level (R² = 0.064, *p* = 0.981) and species level (R² = 0.090, *p* = 0.798).

Alpha diversity was assessed to evaluate within-sample microbial diversity across groups G1–G4. As shown in **Figures 2A, B**, Chao1 richness and Shannon diversity indices exhibited comparable distributions among the four groups, indicating no pronounced differences in overall microbial richness or evenness. Consistently, Kruskal–Wallis analysis showed no significant group-level differences in Chao1 richness (H = 1.099, *p* = 0.777), Shannon diversity (H = 0.519, *p* = 0.915), Simpson index (H = 0.211, *p* = 0.976), or observed species (H = 1.060, *p* = 0.787), with negligible effect sizes (**Supplementary Table 7**). Comparison between the two extreme groups also showed only small effect sizes for these alpha diversity indices (**Supplementary Table 7**).

To further evaluate OSA severity as a continuous measure, Spearman correlation analyses were performed between AHI and alpha diversity indices. AHI was not significantly associated with Chao1 richness (rho = 0.097, *p* = 0.618), Shannon diversity (rho = 0.156, *p* = 0.418), Simpson index (rho = 0.113, *p* = 0.560), or observed species (rho = 0.092, *p* = 0.635; **Supplementary Table 6**). These results indicate that increasing OSA severity was not associated with a monotonic change in global microbial alpha diversity in this cohort.

To assess between-sample community structure, beta diversity was evaluated using Principal Coordinates Analysis (PCoA) based on Bray–Curtis dissimilarity (**Figure 2C**), a widely used metric for comparing microbial community composition (35). Samples from the four groups showed substantial overlap in ordination space, suggesting broadly similar microbial community structures. PERMANOVA based on Bray-Curtis dissimilarity further indicated that OSA severity groups explained only a small proportion of taxonomic variation at both the genus level (R² = 0.064, p = 0.981) and species level (R² = 0.090, p = 0.798; **Supplementary Table 7**). Together, these results suggest that overall gut microbial diversity and community structure did not show strong group-level separation across OSA severity strata, although limited statistical power should be considered.

### 3.3 Functional potential of the gut microbiome

**Figure 3:**
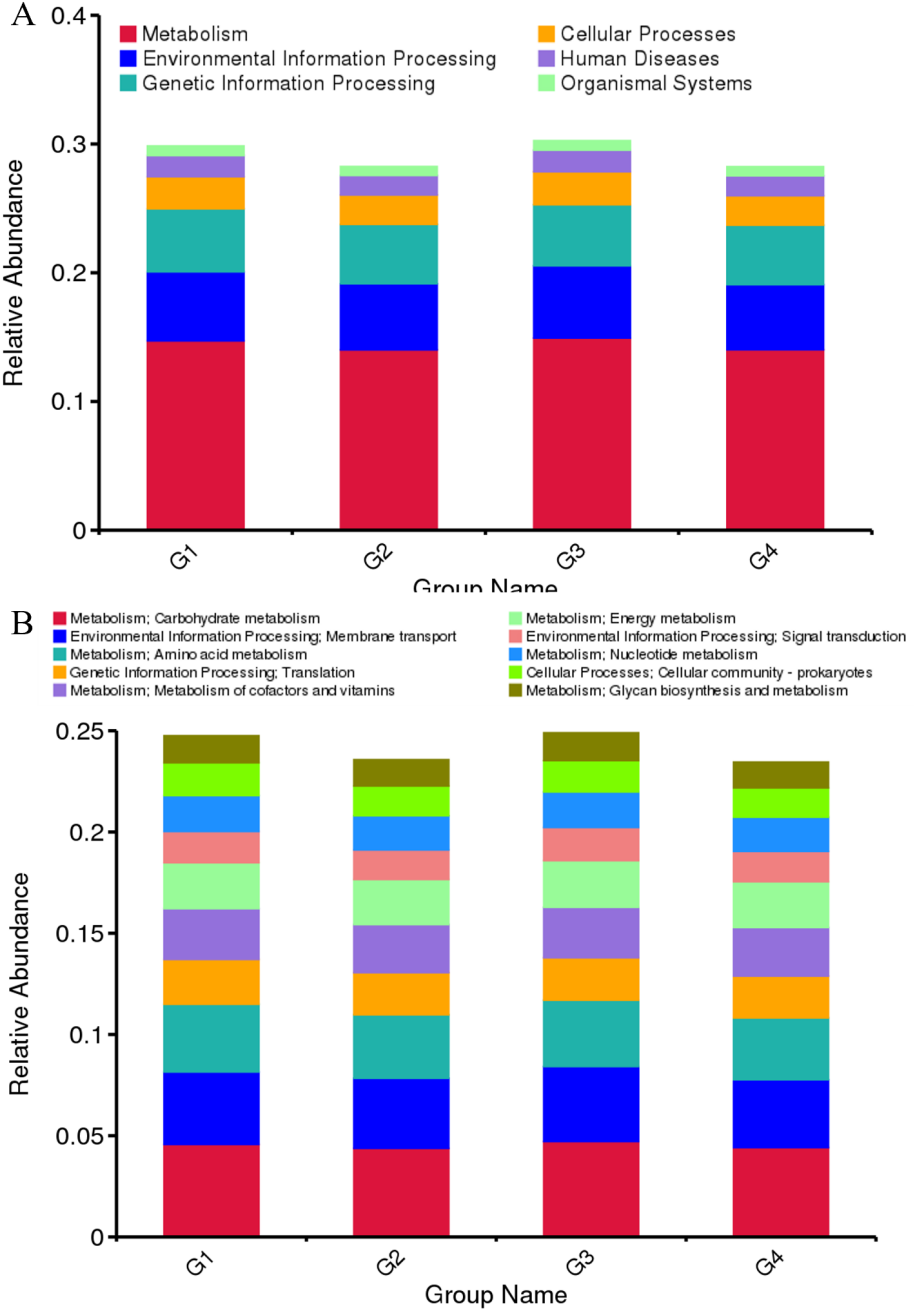
Functional composition of gut microbiota among the four groups based on KEGG annotation. Relative abundance of predicted functional categories at KEGG level 1 **(A)** and level 2 **(B)** is shown for each group (G1–G4). Metabolism represented the dominant functional category across all groups, followed by environmental and genetic information processing. Overall, functional profiles were broadly similar among groups, with moderate variation in specific functional pathways.

To investigate the functional potential of the gut microbiome across groups, functional annotation was performed based on the Kyoto Encyclopedia of Genes and Genomes (KEGG) database. At KEGG level 1, the overall functional composition was highly similar among groups G1 to G4, with metabolism representing the dominant functional category in all samples (**Figure 3A**). Environmental information processing and genetic information processing constituted the next most abundant functional categories, whereas cellular processes, organismal systems, and human disease related functions accounted for smaller proportions.

Consistent functional patterns were also observed at KEGG level 2. As shown in **Figure 3B**, pathways associated with carbohydrate metabolism, amino acid metabolism, energy metabolism, membrane transport, and signal transduction were prevalent across all groups, indicating broadly conserved functional repertoires of the gut microbiome. These functional categories are commonly reported as core components of human gut microbial metabolic capacity (36).

Although moderate variation was observed in the relative abundance of specific level 2 pathways, no strong broad-level functional separation was detected across OSA severity groups at this level of analysis. Detailed functional annotations at KEGG levels 1 to 3 are provided in **Supplementary Table 3** for comprehensive reference. Together, these results suggest that the global functional potential of the gut microbiome remains largely conserved across groups, paralleling the similarity observed in microbial community structure.

### 3.4 Differential microbial taxa between extreme groups

**Figure 4:**
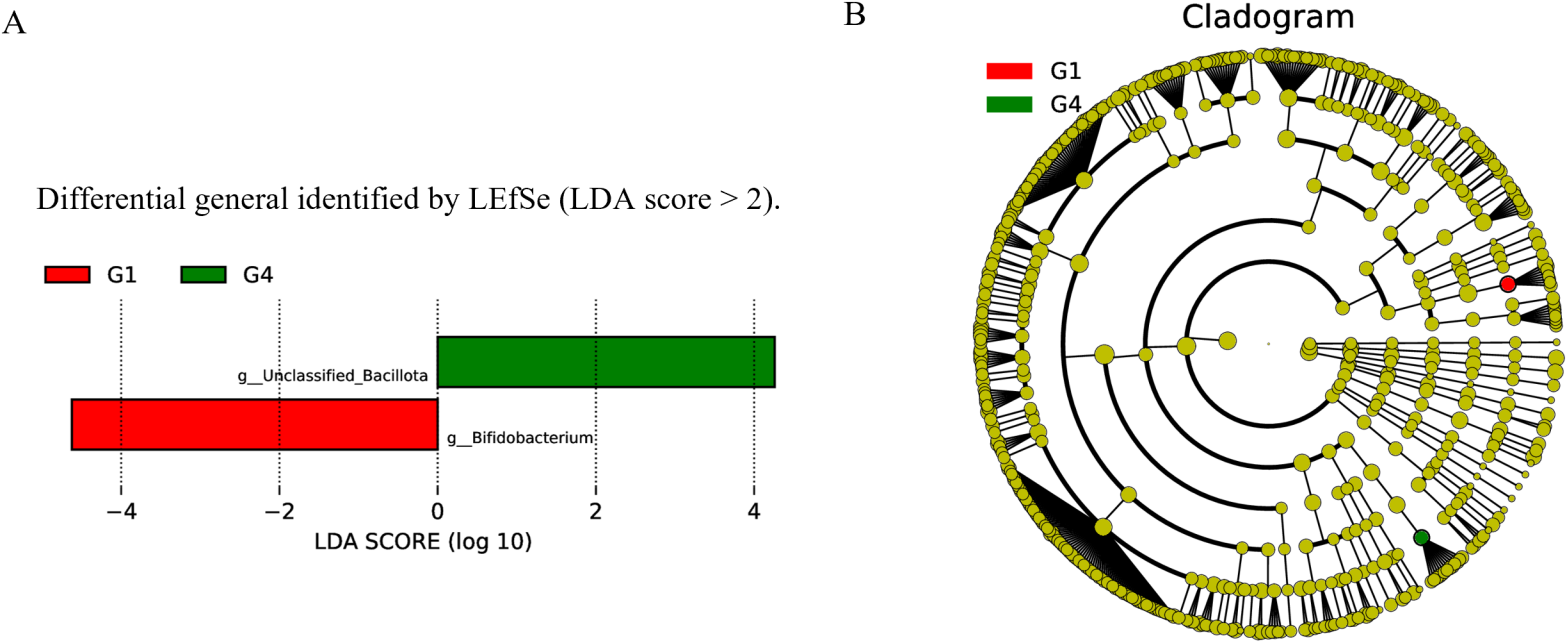
Differential microbial taxa between the two extreme groups identified by LEfSe analysis. **(A)** Differentially abundant taxa between G1 and G4 identified using LEfSe with an LDA score threshold of 2.0. Red bars indicate taxa enriched in G1, while green bars indicate taxa enriched in G4. **(B)** LEfSe cladogram showing the phylogenetic distribution of differentially abundant taxa between G1 and G4 from phylum to genus levels. Nodes highlighted in red represent taxa enriched in G1, whereas nodes in green represent taxa enriched in G4.

To further identify microbial taxa associated with OSA severity, differential taxonomic analysis was performed between the two extreme groups G1 and G4. Linear discriminant analysis effect size (LEfSe) was applied to detect taxa showing consistent differences in relative abundance between the two groups, using an LDA score threshold of 2.0 (**Figure 4A**), as previously described (37).

LEfSe analysis identified multiple taxa that were differentially enriched between G1 and G4 across different taxonomic levels. Specifically, LEfSe analysis identified four discriminative taxa at the genus level with an LDA score greater than 2.0, including two genera enriched in G1 (*Bifidobacterium* and *Escherichia*) and two genera enriched in G4 (*Eubacterium* and *Anaerostipes*) (**Figure 4A**; **Supplementary Table 2**). Several bacterial lineages exhibited higher relative abundance in G1, whereas a distinct set of taxa was enriched in G4, indicating divergent microbial signatures associated with the two extreme clinical phenotypes. The phylogenetic distribution of these differential taxa is illustrated in the cladogram shown in **Figure 4B**, which highlights their hierarchical relationships from phylum to genus levels.

To complement the extreme-group comparison, we further examined whether these representative genera were associated with AHI as a continuous measure of OSA severity. Spearman correlation analysis showed inverse associations between AHI and the relative abundances of Bifidobacterium (rho = -0.551, p = 0.002) and Escherichia (rho = -0.378, p = 0.043), whereas Eubacterium (rho = 0.444, p = 0.016) and Anaerostipes (rho = 0.414, p = 0.025) showed positive associations with AHI (**Supplementary Table 6**). These associations were consistent with the enrichment directions observed in the LEfSe analysis. However, given the exploratory nature of these analyses and the limited sample size, these findings should be interpreted as nominal taxonomic signals rather than definitive OSA severity-associated biomarkers.

These results suggest that, despite the lack of strong group-level separation in overall microbial community structure, selected microbial taxa may vary across the clinical spectrum of OSA severity in PA patients.

### 3.5 Antibiotic resistance gene profiles of the gut microbiota

**Figure 5:**
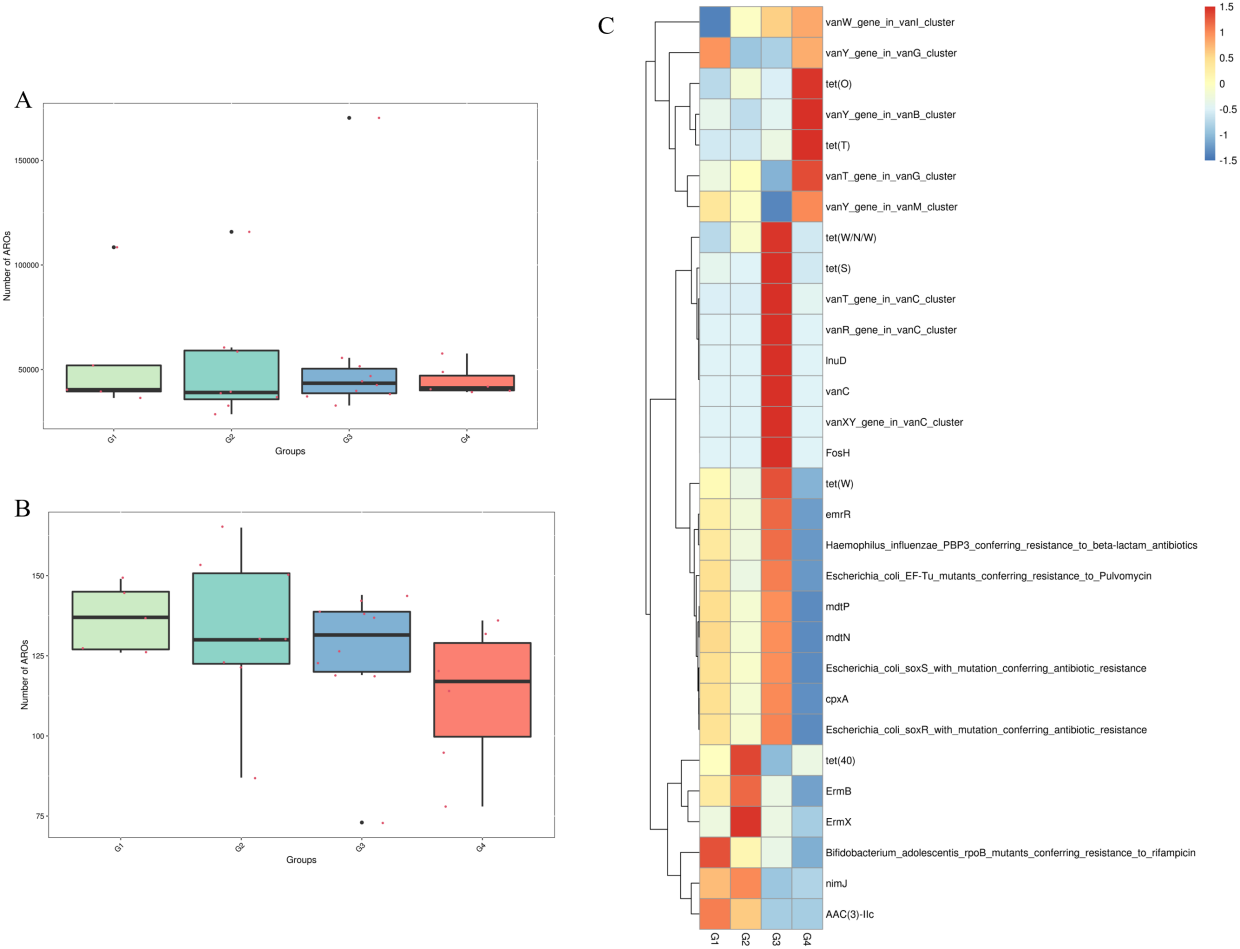
Antibiotic resistance gene (ARG) profiles across groups. **(A)** Comparison of total antibiotic resistance gene (ARG) abundance per sample across groups G1–G4, calculated as the sum of CARD-annotated ARG abundances. Each point represents an individual sample. **(B)** Number of detected antibiotic resistance ontology (ARO) categories per sample across groups, representing ARG richness. **(C)** Heatmap showing relative abundance patterns of representative ARGs annotated using the comprehensive antibiotic resistance database (CARD). Values were z-score normalized across samples prior to hierarchical clustering to visualize similarity patterns.

To characterize antibiotic resistance gene profiles across OSA severity groups, resistome analysis was performed based on annotation against the Comprehensive Antibiotic Resistance Database (CARD). The overall ARG burden was first evaluated by calculating total ARG relative abundance after excluding NonARO features. As shown in **Figure 5A**, total ARG abundance exhibited inter-individual variability across samples but did not differ significantly among groups G1 to G4 (Kruskal–Wallis H = 0.560, *p* = 0.906; **Supplementary Table 9**). Consistently, total ARG relative abundance was not associated with AHI as a continuous measure of OSA severity (rho = 0.007, *p* = 0.973; **Supplementary Table 9**).

In addition to ARG abundance, the diversity of resistance genes was assessed using Antibiotic Resistance Ontology (ARO)-level profiles. The number of detected ARO categories per sample, referred to as ARO richness, showed a decreasing trend from G1 to G4, although the overall group comparison did not reach statistical significance (Kruskal–Wallis H = 4.865, *p* = 0.182; **Figure 5B**; **Supplementary Table 9**). In exploratory continuous-variable analysis, ARO richness was inversely associated with AHI (rho = -0.448, *p* = 0.015; **Supplementary Table 9**). By contrast, ARO Shannon diversity did not differ significantly among groups (H = 1.334, *p* = 0.721) and was not significantly associated with AHI (rho = -0.283, *p* = 0.137; **Supplementary Table 9**).

To further examine resistome composition patterns, a heatmap was constructed based on the relative abundance of representative ARGs annotated at the ARO level (**Figure 5C**). Relative abundance values were z score normalized across samples prior to hierarchical clustering. The resulting heatmap revealed heterogeneous ARG composition patterns across individuals, without distinct group specific clustering. PERMANOVA based on ARO-level Bray–Curtis dissimilarity indicated that OSA severity groups explained only a small proportion of resistome compositional variation (R² = 0.068, *p* = 0.978; **Supplementary Table 9**). Pairwise ANOSIM analyses similarly did not identify significant separation between any two OSA severity groups, including the two extreme groups G1 and G4 (ANOSIM R = 0.037, *p* = 0.291; **Supplementary Table 9**).

To provide further biological context, ARG annotations were summarized according to available CARD resistance mechanism classifications. The dominant resistance mechanism was antibiotic target alteration, followed by antibiotic efflux, antibiotic target protection, and antibiotic inactivation (**Supplementary Table 9**). The most abundant ARO features across samples included vancomycin-related genes, tetracycline resistance genes, macrolide resistance genes, and nitroimidazole resistance genes (**Supplementary Table 9**). Collectively, these results indicate substantial inter-individual variability in the gut resistome of PA patients with coexisting OSA, with no clear OSA severity-specific resistome clustering. The inverse association between ARO richness and AHI should be interpreted as exploratory given the small sample size and potential influence of unmeasured exposures such as prior antibiotic use, diet, and medication history.

### 3.6 Integrated metabolomic analyses of gut microbiota

**Figure 6:**
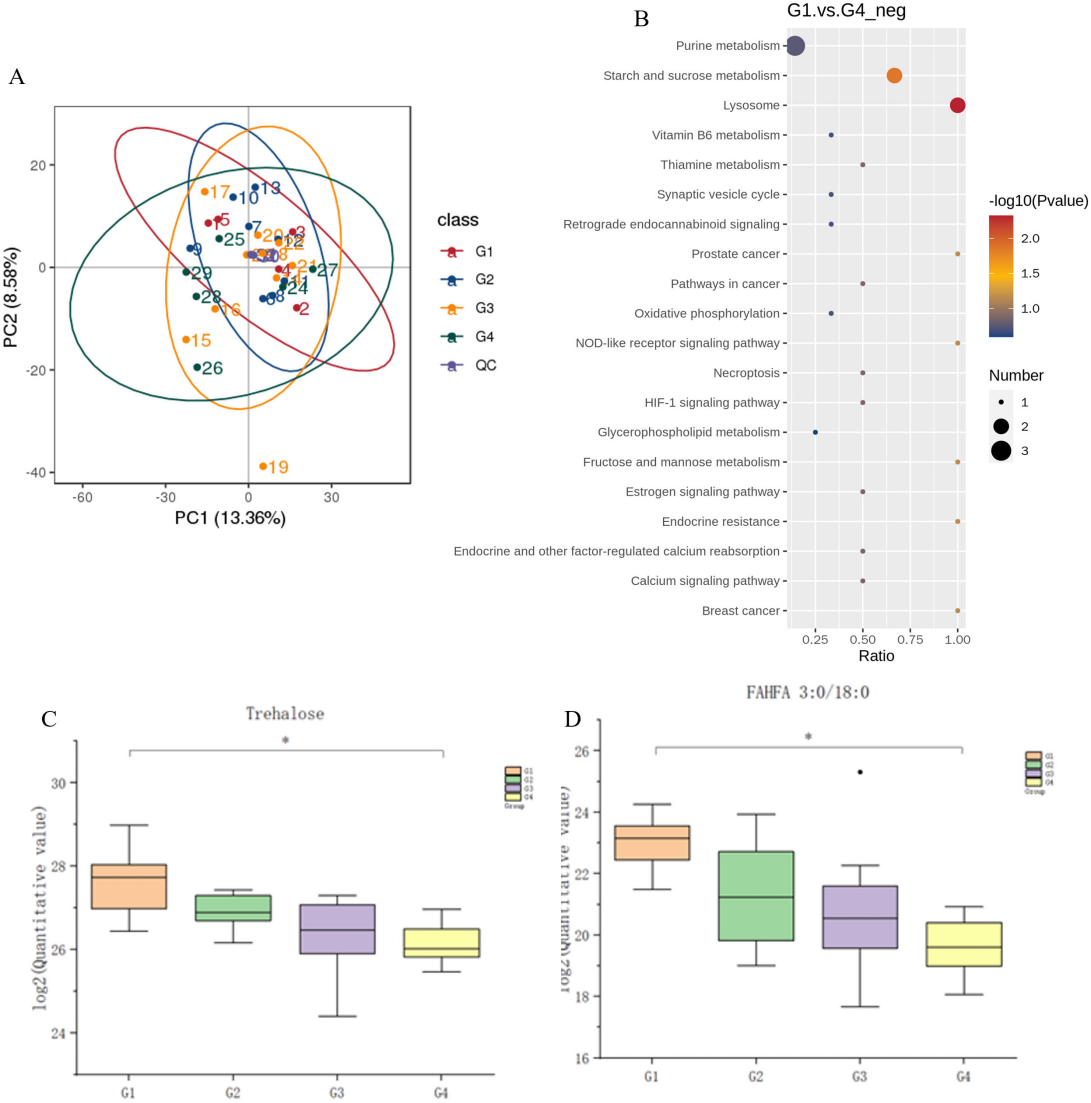
Integrated metabolomic analyses among the four groups. **(A)** PLS-DA score plot based on metabolites detected in the negative ion mode, used for exploratory visualization of metabolic profile variation among groups G1–G4. Quality control (QC) samples clustered tightly, indicating stable analytical performance. **(B)** KEGG pathway enrichment analysis of nominally differential metabolites identified between G1 and G4 in the negative ion mode. **(C, D)** Boxplots of representative differential metabolites across groups, including trehalose **(C)** and FAHFA 3:0/18:0 **(D)**. Values were log2-transformed, and data are presented as box-and-whisker plots with the center line indicating the median.

To further assess metabolic variation associated with clinical stratification, untargeted fecal metabolomic profiling was performed and interpreted alongside the metagenomic results. Multivariate analysis was first conducted to visualize global metabolic variation among groups. Partial least squares discriminant analysis (PLS-DA) was applied as an exploratory approach, revealing partial separation of metabolic profiles among groups G1 to G4, while quality control samples clustered tightly, indicating stable analytical performance (**Figure 6A**). Given the supervised nature of this method, PLS-DA results were interpreted as descriptive visualization rather than definitive evidence of group separation, consistent with established recommendations for metabolomic data analysis (38).

Differential metabolites between G1 and G4 were initially identified based on a combination of variable importance in projection (VIP) scores greater than 1.0 and univariate statistical testing. Using nominal criteria, 53 metabolites in the negative ion mode and 71 metabolites in the positive ion mode showed raw *p* values < 0.05 in the G1 versus G4 comparison. However, after global Benjamini–Hochberg false discovery rate correction across all detected metabolites within each ion mode, no individual metabolite remained significant at FDR < 0.05 (**Supplementary Table 8**). Therefore, metabolite-level differences were interpreted as nominal and exploratory.

To identify metabolic pathways potentially associated with OSA severity, nominally differential metabolites between G1 and G4 were subjected to KEGG pathway enrichment analysis. As shown in **Figure 6B**, several metabolic pathways were enriched among the differential metabolites, indicating pathway-level clustering of the observed metabolic differences. These enrichment results reflect pathway level tendencies rather than direct functional causality, as pathway analysis is sensitive to metabolite coverage, annotation completeness, and the statistical threshold used for defining differential metabolites (39).

Representative differential metabolites were further examined at the individual metabolite level. As illustrated in **Figures 6C, D**, trehalose and FAHFA 3:0/18:0 showed different distributions across groups, with higher levels in G1 than in G4. Exploratory correlation analyses further showed inverse associations between AHI and selected FAHFA species, including FAHFA 2:0/18:0 (rho = -0.577, *p* = 0.001), FAHFA 3:0/18:0 (rho = -0.573, *p* = 0.001), and FAHFA 3:0/18:1 (rho = -0.494, *p* = 0.006), as well as trehalose-related metabolites including α, α-trehalose (rho = -0.563, *p* = 0.001) and trehalose 6-phosphate (rho = -0.456, *p* = 0.013; **Supplementary Table 8**). However, these representative metabolite associations did not survive global FDR correction and should therefore be interpreted as hypothesis-generating.

Overall, fecal metabolomic profiling identified nominal metabolic differences between the two extreme phenotypes and exploratory associations with AHI, whereas global taxonomic diversity and broad functional profiles remained comparatively stable. These results suggest that metabolic readouts may be more sensitive to phenotype-associated variation than broad metagenomic features in this cohort, but the metabolomic findings require validation in larger, independent studies.

## 4 Discussion

### 4.1 Integrated overview and hierarchical pattern of microbiome-associated variation

In this prospective multi-omics study of clinically confirmed primary aldosteronism (PA) with coexisting obstructive sleep apnea (OSA), we observed a layered and exploratory pattern of gut microbiome-associated variation across OSA severity strata. Global microbial gene richness, alpha diversity, beta diversity, and broad KEGG functional profiles did not show strong group-level separation. Additional analyses treating apnea–hypopnea index (AHI) as a continuous variable similarly showed no significant association between AHI and overall gene richness or alpha diversity indices. However, selected microbial taxa, metabolite features, and ARO richness showed exploratory associations with AHI, suggesting that localized microbiome-associated signals may be detectable even when global community-level features remain broadly conserved. These findings support the hypothesis that, in PA with coexisting OSA, clinical heterogeneity may be reflected more clearly by selected taxonomic and metabolic readouts than by overall microbial community structure. Nevertheless, given the small sample size, limited statistical power, and the absence of metabolite-level significance after global false discovery rate correction, these observations should be regarded as hypothesis-generating rather than confirmatory.

Our study should be interpreted in the context of PA as an endocrine disorder with systemic metabolic and cardiovascular consequences. Although OSA is a common comorbidity in patients with PA, the present work was not intended simply as a descriptive analysis of gut microbial variation across sleep-disordered breathing severity. Rather, we used OSA stratification and AHI-based exploratory analyses as a clinically relevant framework to examine which layer of gut microbiome-associated variation may best reflect heterogeneity within PA. From an endocrine perspective, the findings suggest that host-microbiome interactions in this comorbidity may be more readily reflected at the metabolic interface than at the level of overall gut community composition.

Across the four clinical groups, metagenomic analyses indicated relative stability of global community properties. Gene richness and alpha diversity indices were comparable among groups, and Bray–Curtis PERMANOVA showed that OSA severity groups explained only a small proportion of taxonomic variation at both the genus and species levels. These results are consistent with the view that clinically relevant heterogeneity in chronic cardiometabolic and endocrine-related conditions may occur without large-scale disruption of overall microbial diversity (25, 26). However, the lack of statistically significant differences should not be interpreted as evidence of biological equivalence, because the small sample size substantially limits statistical power and may obscure subtle effects.

Despite this apparent stability at the global community level, more refined analyses revealed selective signals. LEfSe analysis identified specific genera that differed between the two extreme groups, and the same genera showed exploratory associations with AHI. In parallel, fecal metabolomic profiling identified nominal metabolic differences between G1 and G4, including trehalose-related metabolites and FAHFA species that were inversely associated with AHI. These findings are compatible with ecological principles in which microbial communities maintain compositional robustness through buffering and functional redundancy, allowing different taxa to encode overlapping metabolic capacities and preserve core functions (40, 41). Under such conditions, changes in microbial activity, host–microbe interactions, or downstream metabolic outputs may not be fully captured by alpha diversity, beta diversity, or broad gene-centric functional summaries alone.

Metabolites represent integrated biochemical outputs of microbial metabolism, host physiology, and environmental inputs, and therefore may provide a sensitive readout of functional variation at the host-microbiome interface (10). In the context of endocrine dysregulation and sleep-related intermittent hypoxia, metabolic outputs may capture aspects of disease-associated heterogeneity that are not apparent from global microbial community structure. However, in the present study, no individual metabolite remained significant after global Benjamini–Hochberg correction across all detected metabolites. Therefore, the metabolomic findings should be interpreted as exploratory pathway- and metabolite-level tendencies rather than validated biomarkers or mechanistic evidence.

Taken together, our results suggest a hierarchical pattern of microbiome-associated variation in PA with coexisting OSA: global microbial structure and broad functional potential appeared relatively conserved, selected taxa and resistome richness showed exploratory associations with AHI, and fecal metabolomic profiles showed nominal phenotype-associated variation. This layered pattern provides a framework for future studies but requires validation in larger, controlled cohorts with more comprehensive clinical, dietary, medication, and PA subtype information.

### 4.2 Selective taxonomic signals and their potential biological relevance

Although global gut microbial diversity and overall taxonomic structure did not show strong separation across OSA severity strata, several taxa differed between the two extreme groups and showed exploratory associations with AHI. Specifically, Bifidobacterium and Escherichia were enriched in G1 and negatively associated with AHI, whereas Eubacterium and Anaerostipes were enriched in G4 and positively associated with AHI. This pattern supports the view that PA with coexisting OSA may not be characterized by broad dysbiosis at the community level, but rather by selective taxonomic variation embedded within a relatively stable global microbial background.

The observed association involving *Bifidobacterium* may be relevant because this genus is commonly considered a beneficial commensal group with roles in carbohydrate fermentation, epithelial barrier function, and immune modulation. In the context of OSA, intermittent hypoxia and sleep fragmentation have been linked to gut dysbiosis, impaired barrier function, inflammation, and altered production of short-chain fatty acids (SCFAs), which are important mediators of host metabolic and immune homeostasis (42, 43). Recent studies and reviews further suggest that OSA-related hypoxia burden may be associated with reductions in selected SCFA-producing bacteria and altered gut microbial structure (44, 45). Therefore, the inverse association between Bifidobacterium and AHI may reflect a reduction in potentially protective microbial features with increasing OSA severity, although this interpretation remains speculative.

The positive associations of *Eubacterium* and *Anaerostipes* with AHI are more complex. Members of these genera can include anaerobic fermenters involved in SCFA production, including butyrate-related metabolic pathways, and their biological roles may vary depending on species, strain, substrate availability, and host context. Therefore, enrichment of these genera in the severe OSA group should not be interpreted simply as beneficial or harmful. Instead, it may indicate remodeling of anaerobic carbohydrate fermentation networks or compensatory shifts in the gut ecosystem under conditions of OSA-related physiological stress. This is consistent with the broader concept that microbial functional redundancy may preserve global community-level features while allowing selective taxa to shift in response to host or environmental pressures.

The finding involving *Escherichia* should also be interpreted cautiously. Although some *Escherichia* members are commonly associated with facultative anaerobic expansion and inflammatory dysbiosis, genus-level metagenomic annotation does not distinguish pathogenic from commensal strains. In our cohort, *Escherichia* was enriched in the lower OSA severity group and showed an inverse association with AHI, which does not support a simple interpretation of progressive inflammatory dysbiosis with OSA severity. This result may reflect inter-individual variability, strain-level heterogeneity, or the limited sample size rather than a disease-specific pattern.

Overall, the taxonomic findings suggest that selected genera may track aspects of OSA severity more sensitively than broad alpha or beta diversity metrics. However, because these associations were exploratory, based on a small cohort, and not adjusted for detailed dietary intake, medication exposure, or PA subtype, they should be interpreted as hypothesis-generating. Future studies integrating strain-level profiling, metatranscriptomics, targeted SCFA quantification, and detailed clinical covariates will be needed to clarify whether these taxa contribute mechanistically to PA–OSA heterogeneity or simply reflect downstream ecological variation.

### 4.3 Metabolomic variation and interpretation of FAHFA and trehalose-related signals

Fecal metabolomic profiling showed more detectable phenotype-associated variation than global metagenomic diversity or broad KEGG functional profiles. However, this finding should be interpreted cautiously. Although nominally differential metabolites and pathway-level enrichment patterns were observed between G1 and G4, no individual metabolite remained significant after global Benjamini–Hochberg false discovery rate correction across all detected metabolites. Therefore, the metabolomic findings in this study should be viewed as exploratory metabolic signals rather than validated biomarkers.

Among the representative metabolites examined, several FAHFA species showed higher levels in G1 than in G4 and inverse associations with AHI. FAHFAs are a class of endogenous lipids composed of fatty acids esterified to hydroxy fatty acids. Since their initial characterization, FAHFAs have attracted attention because selected members, particularly PAHSA species, have been linked to glucose homeostasis, insulin sensitivity, and anti-inflammatory activity (46, 47). In the present cohort, lower fecal FAHFA-related signals in the severe OSA group may suggest altered lipid-related metabolic readouts in PA patients with greater sleep-disordered breathing severity. This observation is biologically plausible given the close relationships among PA, OSA, inflammation, insulin resistance, and cardiometabolic risk. Nevertheless, FAHFAs constitute a structurally diverse lipid class, and biological activities can vary substantially according to acyl chain composition and isomeric structure (46). Therefore, the observed FAHFA associations should not be interpreted as evidence of a specific protective or pathogenic mechanism without targeted lipidomic validation.

Trehalose-related metabolites also showed nominal differences between the two extreme groups and inverse exploratory associations with AHI. Trehalose is a naturally occurring non-reducing disaccharide found in bacteria, fungi, plants, and invertebrates, and it can function as an energy source, osmoprotectant, and stress-response molecule in many organisms (48, 49). In the gut ecosystem, trehalose availability may influence microbial carbohydrate utilization and community structure. Experimental work using human gut model systems has shown that trehalose supplementation can remodel microbiota composition and microbial metabolic activity, although its effects may be context-dependent (50). Therefore, reduced trehalose-related signals with increasing AHI may reflect changes in microbial carbohydrate metabolism, dietary substrate availability, or host–microbiome metabolic exchange in severe OSA.

These metabolomic observations may help explain why metabolic readouts appeared more sensitive than broad metagenomic features in this cohort. Metabolites integrate signals from microbial activity, host physiology, diet, and environmental exposures; therefore, they may capture functional variation that is not apparent from community composition alone. However, the same integrative nature also makes fecal metabolomic profiles vulnerable to confounding by dietary intake, medication exposure, bowel habits, and other unmeasured environmental factors. Because these variables were not comprehensively captured in the present study, the observed FAHFA and trehalose-related findings should be interpreted as hypothesis-generating. Future studies should include targeted quantification of FAHFA species, trehalose-related metabolites, short-chain fatty acids, and inflammatory or metabolic host markers to clarify whether these signals are mechanistically linked to PA–OSA pathophysiology.

### 4.4 Resistome features and inter-individual variability

In addition to taxonomic and metabolomic profiling, we characterized gut resistome features based on CARD annotations. Overall ARG relative abundance did not differ significantly across OSA severity groups and was not associated with AHI. ARO-level Bray–Curtis PERMANOVA and pairwise ANOSIM analyses also showed no clear group-level separation in resistome composition. These findings suggest that, within this cohort, OSA severity was not associated with a distinct resistome signature at the level of overall ARG burden or ARO composition.

However, ARO richness showed an exploratory inverse association with AHI, suggesting that the number of detected resistance ontology categories may decrease with increasing OSA severity. This finding should be interpreted cautiously. ARO richness can be influenced by sequencing depth, microbial community composition, prior antibiotic exposure, diet, geography, environmental exposures, and medication history. Because detailed information on antibiotic exposure history, diet, and medication use was not available beyond the exclusion of recent antibiotics or probiotics before fecal sampling, we cannot determine whether the observed ARO richness trend reflects OSA-related biology, PA-related host factors, or unmeasured environmental and clinical exposures.

The lack of distinct group-specific resistome clustering is not unexpected. The human gut microbiota constitutes a large reservoir of ARGs, and gut resistome profiles are known to show substantial inter-individual and population-level variability (18, 19, 51). Prior metagenomic studies have shown that resistome composition may reflect antibiotic exposure patterns, geography, diet, animal-associated antibiotic use, and other environmental factors, rather than a single disease phenotype alone (18, 19). In this context, the marked inter-individual variability observed in our cohort may have masked subtle disease-severity-associated signals.

At the resistance-mechanism level, ARG annotations were dominated by antibiotic target alteration, followed by antibiotic efflux, antibiotic target protection, and antibiotic inactivation. These broad mechanisms are commonly represented in gut metagenomic resistome datasets and may reflect the composite contribution of diverse commensal taxa rather than expansion of a single resistance-associated lineage. The most abundant ARO features included vancomycin-related genes, tetracycline resistance genes, macrolide resistance genes, and nitroimidazole resistance genes. However, because these features did not form a clear OSA severity-specific pattern, their clinical significance in PA with coexisting OSA remains uncertain.

Mobile genetic elements (MGEs) are important vehicles for ARG dissemination through horizontal gene transfer and can shape the mobility potential of gut resistomes (52, 53). Although MGE-related annotations were available in the metagenomic output, the present study was not designed to perform robust ARG–MGE co-localization or strain-resolved transfer analysis. Therefore, we did not infer ARG mobility or transmission potential from the available data. Future studies integrating long-read sequencing, metagenome-assembled genomes, plasmid reconstruction, and strain-level analysis will be needed to clarify whether ARGs identified in PA–OSA patients are linked to mobile elements or specific bacterial hosts.

Overall, the resistome results indicate substantial inter-individual variability without a clear OSA severity-specific ARG composition. The exploratory inverse association between ARO richness and AHI may warrant further investigation, but it should be interpreted as hypothesis-generating given the small sample size and incomplete control of antibiotic, dietary, environmental, and medication-related confounders.

### 4.5 Clinical confounders and limitations of interpretation

Several limitations should be considered when interpreting the present findings. First, the study included only 29 patients, and the sample size of each OSA severity subgroup was small. This substantially limited statistical power, particularly for detecting modest microbiome or metabolome differences across four clinical strata. Therefore, the absence of significant differences in gene richness, alpha diversity, beta diversity, broad functional profiles, and resistome composition should not be interpreted as evidence of biological equivalence. To partially address this concern, we supplemented group-based analyses with effect size estimates and exploratory analyses treating AHI as a continuous variable. Nevertheless, these analyses remain exploratory and require validation in larger cohorts.

Second, several clinically important confounders could not be fully addressed. Diet, medication exposure, bowel habits, lifestyle factors, and prior antibiotic exposure can strongly influence gut microbial composition, fecal metabolomic profiles, and resistome features. In the present study, patients with recent use of antibiotics, probiotics, or other microbiota-altering medications within one month before fecal sampling were excluded. However, detailed dietary intake, long-term antibiotic exposure history, antihypertensive medication use, mineralocorticoid receptor antagonist exposure, and other lifestyle-related variables were not systematically captured in the finalized multi-omics analytical dataset. These unmeasured factors may have contributed to the inter-individual variability observed in the microbiome, metabolome, and resistome data.

Third, PA-related clinical heterogeneity was incompletely characterized. Although all participants had clinically confirmed PA and underwent confirmatory testing using the recumbent saline infusion test, individual-level post-suppression aldosterone concentrations and PA subtype information, such as unilateral versus bilateral PA or aldosterone-producing adenoma versus bilateral adrenal hyperplasia, were not available for integrated microbiome–metabolome analysis. Because PA subtype and aldosterone burden may influence metabolic risk, cardiovascular phenotype, and potentially host–microbiome interactions, the lack of subtype-stratified analyses limits the endocrine interpretation of the present findings. Future studies should incorporate post-suppression aldosterone concentrations, adrenal venous sampling or imaging-based subtype information, and treatment status to clarify whether microbiome-associated readouts differ according to PA subtype or aldosterone burden.

Fourth, the metabolomic findings require cautious interpretation. Although nominal metabolite-level differences and pathway enrichment patterns were observed between the two extreme groups, no individual metabolite remained significant after global false discovery rate correction across all detected metabolites. Therefore, the observed FAHFA- and trehalose-related associations should be considered hypothesis-generating rather than validated metabolic biomarkers. Targeted metabolomic validation, absolute quantification, and independent replication will be necessary to determine whether these metabolites are reproducibly associated with PA–OSA heterogeneity.

Fifth, the resistome analysis was based on CARD annotations and provided useful ARO-level and resistance-mechanism summaries, but it did not establish ARG mobility, expression, or clinical resistance phenotype. MGE-related annotations were available, but robust ARG–MGE co-localization analysis, strain-level host assignment, and plasmid reconstruction were beyond the scope of the present study. Therefore, the resistome results should be interpreted as ecological descriptors of gut ARG profiles rather than evidence of clinically transmissible resistance.

Finally, the observational and cross-sectional design precludes causal inference. It remains unclear whether the observed taxonomic, metabolic, and resistome features contribute to PA–OSA pathophysiology, reflect downstream consequences of endocrine and sleep-related physiological stress, or are shaped by external exposures. Longitudinal studies incorporating dietary records, medication history, PA subtype, post-suppression aldosterone concentrations, targeted metabolomics, and mechanistic validation will be needed to determine whether gut microbiome-associated signals have diagnostic, prognostic, or therapeutic relevance in PA with coexisting OSA.

## 5 Conclusion

This exploratory multi-omics study suggests that, in patients with primary aldosteronism and coexisting obstructive sleep apnea, global gut microbial diversity and broad functional potential remain relatively conserved across OSA severity strata, whereas selected microbial taxa, fecal metabolic features, and resistome-related indices may show more detectable associations with AHI. However, because metabolite-level findings did not survive global FDR correction and the study was limited by small sample size and incomplete control of dietary, medication, and PA subtype-related confounders, these results should be interpreted as hypothesis-generating. Larger controlled and longitudinal studies incorporating PA subtype, post-suppression aldosterone concentrations, medication exposure, dietary assessment, and targeted metabolomic validation are needed to clarify the role of gut microbiome-associated readouts in PA–OSA heterogeneity.

## Data Availability

The original contributions presented in the study are included in the article/**Supplementary Material**. Further inquiries can be directed to the corresponding author.

## Supplementary material

Supplementary Table 1 Baseline clinical characteristics of the study participants. Data are presented as mean ± standard deviation or number (n). Differences among groups were assessed using one-way ANOVA for continuous variables and chi-square test for categorical variables. A p value < 0.05 was considered statistically significant. PAC, plasma aldosterone concentration; PRA, plasma renin activity; ARR, aldosterone-to-renin ratio; K+, serum potassium; GLU, fasting glucose; CHOL, total cholesterol; TG, triglycerides; LDL, low-density lipoprotein cholesterol; HDL, high-density lipoprotein cholesterol; 24h USP, 24-hour urinary sodium excretion; 24h UPP, 24-hour urinary potassium excretion; BMI, body mass index.

Supplementary Table 2 Differential genera identified between G1 and G4 by LEfSe analysis.

Supplementary Table 3 KEGG functional profiles of gut microbiota at levels 1, 2, and 3. Relative abundances of KEGG pathways were calculated based on annotated unigenes and normalized within each group (sum = 1). At each KEGG level, only the top pathways are shown, and the remaining pathways are grouped as “Others”. G1–G4 represent primary aldosteronism patients without OSA (G1) and with mild (G2), moderate (G3), and severe OSA (G4), respectively.

Supplementary Table 4 KEGG pathway enrichment analysis of differential metabolites between G1 and G4 (negative ion mode).

Supplementary Table 5 Abundance of antibiotic resistance genes (ARGs) across the four groups. ARGs were annotated using the Comprehensive Antibiotic Resistance Database (CARD). Values represent the relative abundance of antibiotic resistance ontology (ARO) entries in each group (G1–G4).

Supplementary Figure 1 Taxonomic composition of gut microbiota at phylum and genus level. **(A)** Relative abundance of dominant bacterial phyla across groups G1–G4. **(B)** Relative abundance of dominant bacterial genera across groups G1–G4. Taxa with low relative abundance were grouped as “Others”.
