# Supplementary material for: Metagenomic and metabolomic profiling in primary aldosteronism with coexisting obstructive sleep apnea
Source: Front Endocrinol (Lausanne). 2026 Jul 20;17:1858100. doi: 10.3389/fendo.2026.1858100 (PMC13429393; doi:10.3389/fendo.2026.1858100)
Supplement: Supplementary Table 4 — KEGG pathway enrichment analysis of differential metabolites between G1 and G4 (negative ion mode). [file Table4.docx]

Table S1. Baseline clinical characteristics of the study participants. Data are presented as mean ± standard deviation, median (interquartile range), or number/proportion, as appropriate. Differences among groups were assessed using one-way ANOVA or Kruskal–Wallis test for continuous variables, and chi-square test or Fisher’s exact test for categorical variables, as appropriate. A *p* value < 0.05 was considered statistically significant.

PAC, plasma aldosterone concentration; PRA, plasma renin activity; ARR, aldosterone-to-renin ratio; K⁺, serum potassium; GLU, fasting glucose; CHOL, total cholesterol; TG, triglycerides; LDL, low-density lipoprotein cholesterol; HDL, high-density lipoprotein cholesterol; AHI, apnea–hypopnea index; 24h SBP, 24-hour systolic blood pressure; 24h DBP, 24-hour diastolic blood pressure; BMI, body mass index.

| Variable | G1  (n=5) | G2  (n=8) | G3  (n=10) | G4  (n=6) | *p* value |
| --- | --- | --- | --- | --- | --- |
| Sex (male/female) | 1/4 | 4/4 | 5/5 | 5/1 | 0.261 |
| Age (years) | 45.40±13.58 | 51.13±13.67 | 48.10±14.04 | 44.33±8.80 | 0.771 |
| BMI (kg/m^2^) | 26.60±5.32 | 24.95±4.37 | 25.53±4.00 | 28.14±4.30 | 0.567 |
| PAC (ng/dL) | 0.13±0.10 | 0.19±0.09 | 0.16（0.08,0.19） | 0.27±0.16 | 0.321 |
| PRA (ng/mL/hr) | 17.47±9.90 | 22.54±13.66 | 19.85（14.70,31） | 18.24±7.00 | 0.820 |
| ARR | 207.03±195.56 | 133.61±69.13 | 195.98±121.26 | 64.43（49.24,152.71） | 0.368 |
| K^+^ (mmol/L) | 3.88±0.36 | 3.71±0.49 | 3.49±0.30 | 3.61±0.55 | 0.380 |
| GLU (mmol/L) | 4.80±0.36 | 4.71±0.67 | 5.00（4.59,5.67） | 4.98（4.47,7.43） | 0.425 |
| CHOL (mmol/L) | 4.20±0.99 | 4.35±0.68 | 4.17±0.87 | 4.45±1.19 | 0.934 |
| TG (mmol/L) | 1.13±0.26 | 1.75±1.2 | 1.87±0.96 | 1.81±1.28 | 0.609 |
| LDL (mmol/L) | 2.66 ± 0.65 | 2.56 ± 0.47 | 2.34 ± 0.67 | 2.71 ± 0.85 | 0.983 |
| HDL (mmol/L) | 1.04±0.12 | 1.11±0.24 | 1.07±0.25 | 1.11±0.58 | 0.983 |
| AHI | 3.26±1.98 | 8.56±2.50 | 20.56±4.12 | 56.35±21.91 | <0.0001 |
| 24-h SBP (mmHg) | 131,60±5.77 | 144.13±11.29 | 142.30±14.76 | 146.5±13.38 | 0.239 |
| 24-h DBP (mmHg) | 86.80±5.45 | 97.88±12.60 | 91.40±19.960 | 95.67±5.68 | 0.532 |
| Coronary heart disease (n) | 0/5 | 0/8 | 2/8 | 0/6 | 0.414 |
| Diabetes (n) | 0/5 | 0/8 | 2/8 | 0/6 | 0.414 |

Table S2. Differential genera identified between G1 and G4 by LEfSe analysis.

| Taxonomic level | Taxon | Enriched group | LDA score | p value |
| --- | --- | --- | --- | --- |
| Genus | *Bifidobacterium* | G1 | 4.92 | 0.0446 |
| Genus | *Eubacterium* | G4 | 4.72 | 0.028 |
| Genus | *Anaerostipes* | G4 | 4.67 | 0.011 |
| Genus | *Escherichia* | G1 | 4.96 | 0.0446 |

Table S6. AHI continuous association analysis. Spearman correlations; selected genus results are exploratory and nominal.

| Layer | Feature | Spearman rho with AHI | *p* value (AHI) | Spearman rho with mean O_2_ saturation | *p* value (mean O_2_) |
| --- | --- | --- | --- | --- | --- |
| Global metagenomic feature | gene number | 0.104 | 0.589 | -0.132 | 0.494 |
| Global metagenomic feature | ACE | 0.085 | 0.662 | -0.097 | 0.617 |
| Global metagenomic feature | Chao1 | 0.097 | 0.618 | -0.074 | 0.703 |
| Global metagenomic feature | Shannon | 0.156 | 0.418 | -0.107 | 0.580 |
| Global metagenomic feature | Simpson | 0.113 | 0.560 | -0.059 | 0.759 |
| Global metagenomic feature | Observed species | 0.092 | 0.635 | -0.099 | 0.609 |
| Selected genus | Bifidobacterium | -0.551 | 0.002 | -0.075 | 0.699 |
| Selected genus | Escherichia | -0.378 | 0.043 | 0.246 | 0.197 |
| Selected genus | Eubacterium | 0.444 | 0.0159 | -0.160 | 0.406 |
| Selected genus | Anaerostipes | 0.414 | 0.025 | -0.270 | 0.155 |

Table S7. Diversity effect sizes and PERMANOVA.

| Feature | Kruskal-Wallis H | *p* value | epsilon squared | G1 median | G4 median | G1 vs G4 Cliff delta | G1 vs G4 Mann-Whitney *p* |
| --- | --- | --- | --- | --- | --- | --- | --- |
| gene number | 1.817816092 | 0.611 | 0 | 235035 | 327574.5 | -0.2 | 0.662 |
| ACE | 0.97091954 | 0.808 | 0 | 2157.692 | 2683.958 | -0.133 | 0.792 |
| Chao1 | 1.099310345 | 0.778 | 0 | 2173.447 | 2701.836 | -0.2 | 0.662 |
| Shannon | 0.518505747 | 0.915 | 0 | 6.700 | 6.829 | -0.267 | 0.537 |
| Simpson | 0.210574713 | 0.976 | 0 | 0.966 | 0.965 | -0.133 | 0.792 |
| Observed species | 1.059655172 | 0.787 | 0 | 2107 | 2618 | -0.2 | 0.662 |

Table S8. Metabolite FDR and representative correlations.

Table S8A. Global FDR correction of G1 vs. G4 metabolite comparisons.

| Ion mode | Total detected metabolites | Metabolites with raw *p* <0.05 | Metabolites with global BH-FDR <0.05 |
| --- | --- | --- | --- |
| Negative ion mode | 717 | 53 | 0 |
| Positive ion mode | 1,291 | 71 | 0 |

Table S8B. Representative metabolites showing nominal differences between G1 and G4 and exploratory associations with AHI.

| Ion mode | Negative | Negative | Negative | Negative | Negative | Positive |
| --- | --- | --- | --- | --- | --- | --- |
| Metabolite ID | Com_5876_neg | Com_2465_neg | Com_581_neg | Com_54847_neg | Com_3391_neg | Com_353_pos |
| Metabolite name | FAHFA 2:0/18:0 | FAHFA 3:0/18:0 | FAHFA 3:0/18:1 | α,α-Trehalose | Trehalose 6-phosphate | Trehalose |
| FC, G1/G4 | 10.27 | 23.16 | 14.47 | 2.10 | 5.16 | 3.33 |
| Raw *p* value | 0.000599 | 0.000755 | 0.00512 | 0.0186 | 0.0439 | 0.00535 |
| Global BH-FDR | 0.214 | 0.214 | 0.496 | 0.649 | 0.656 | 0.791 |
| Spearman rho with AHI | -0.577 | -0.573 | -0.494 | -0.563 | -0.456 | -0.366 |
| *p* value for AHI correlation | 0.00104 | 0.00115 | 0.00643 | 0.00149 | 0.0130 | 0.0506 |
| Spearman rho with mean O_2_ saturation | 0.024 | 0.074 | -0.057 | 0.136 | -0.040 | -0.174 |
| *p* value for mean O_2_ saturation correlation | 0.903 | 0.702 | 0.770 | 0.483 | 0.835 | 0.368 |

Note: FC indicates the fold change of metabolite intensity in G1 relative to G4. Global Benjamini–Hochberg false discovery rate correction was applied across all detected metabolites within each ion mode. Although several metabolites showed nominal differences and exploratory correlations with AHI, none remained significant after global FDR correction.

Table S9. Antibiotic resistance gene profiles and CARD-based resistome analyses.

Table S9A. ARG abundance, ARO richness, and ARO diversity across OSA severity groups.

| ARG metric | Total ARG relative abundance | ARO richness | ARO Shannon diversity |
| --- | --- | --- | --- |
| Kruskal–Wallis H | 0.560 | 4.865 | 1.334 |
| Group comparison p value | 0.906 | 0.182 | 0.721 |
| Epsilon squared | 0.000 | 0.075 | 0.000 |
| G1 median | 0.00173 | 137 | 3.230 |
| G4 median | 0.00176 | 117 | 3.056 |
| G1 vs. G4 Cliff’s delta | -0.067 | 0.733 | 0.467 |
| G1 vs. G4 Mann–Whitney *p* value | 0.931 | 0.052 | 0.247 |
| Spearman rho with AHI | 0.007 | -0.448 | -0.283 |
| *p* value for AHI correlation | 0.973 | 0.0147 | 0.137 |
| Spearman rho with mean oxygen saturation | 0.135 | 0.371 | 0.255 |
| *p* value for mean oxygen saturation correlation | 0.484 | 0.0474 | 0.182 |

Table S9B. PERMANOVA analysis of ARO-level resistome composition.

| Analysis | F statistic | R^2^ | *p* value |
| --- | --- | --- | --- |
| ARO-level Bray-Curtis PERMANOVA | 0.610 | 0.068 | 0.978 |

Table S9C. Pairwise ANOSIM analysis of ARO-level resistome composition.

| Comparison | ANOSIM R | *p* value |
| --- | --- | --- |
| G1 vs. G2 | -0.099 | 0.855 |
| G1 vs. G3 | -0.012 | 0.426 |
| G1 vs. G4 | 0.037 | 0.291 |
| G2 vs. G3 | -0.038 | 0.723 |
| G2 vs. G4 | -0.059 | 0.758 |
| G3 vs. G4 | -0.086 | 0.763 |

Table S9D. Top ARO features by mean relative abundance across all samples.

| Rank | ARO feature | Mean relative abundance |
| --- | --- | --- |
| 1 | vanT_gene_in_vanG_cluster | 2.08 × 10⁻⁴ |
| 2 | vanY_gene_in_vanB_cluster | 1.48 × 10⁻⁴ |
| 3 | tet(O) | 1.47 × 10⁻⁴ |
| 4 | ErmB | 1.29 × 10⁻⁴ |
| 5 | vanW_gene_in_vanI_cluster | 1.27 × 10⁻⁴ |
| 6 | nimJ | 9.23 × 10⁻⁵ |
| 7 | tet(T) | 8.07 × 10⁻⁵ |
| 8 | tet(40) | 6.01 × 10⁻⁵ |
| 9 | vanH_gene_in_vanB_cluster | 5.14 × 10⁻⁵ |
| 10 | vanY_gene_in_vanM_cluster | 4.68 × 10⁻⁵ |
| 11 | vanG | 4.44 × 10⁻⁵ |
| 12 | tet(32) | 4.16 × 10⁻⁵ |
| 13 | AAC(6')-Ie-APH(2'')___Ia_bifunctional_protein | 3.67 × 10⁻⁵ |
| 14 | tet(W) | 3.63 × 10⁻⁵ |
| 15 | dfrF | 3.53 × 10⁻⁵ |

Table S9E. CARD resistance mechanism summary.

| Resistance mechanism | Total count |
| --- | --- |
| Antibiotic target alteration | 890 |
| Antibiotic efflux | 146 |
| Antibiotic target protection | 81 |
| Antibiotic inactivation | 62 |
| Antibiotic target replacement | 8 |
| Antibiotic target alteration and antibiotic target replacement | 4 |
| Antibiotic efflux and reduced permeability to antibiotic | 4 |
| Antibiotic efflux and antibiotic target alteration | 2 |
| Antibiotic efflux, antibiotic target alteration, and reduced permeability to antibiotic | 2 |
| Reduced permeability to antibiotic | 2 |

Note: ARG, antibiotic resistance gene; ARO, Antibiotic Resistance Ontology. Total ARG relative abundance was calculated after excluding NonARO features. PERMANOVA was performed based on Bray–Curtis dissimilarity of ARO-level profiles. Resistance mechanisms were summarized according to available CARD mechanism annotations.
