# Supplementary figures and images for: Metagenomic and metabolomic profiling in primary aldosteronism with coexisting obstructive sleep apnea
Source: Front Endocrinol (Lausanne). 2026 Jul 20;17:1858100. doi: 10.3389/fendo.2026.1858100 (PMC13429393; doi:10.3389/fendo.2026.1858100)

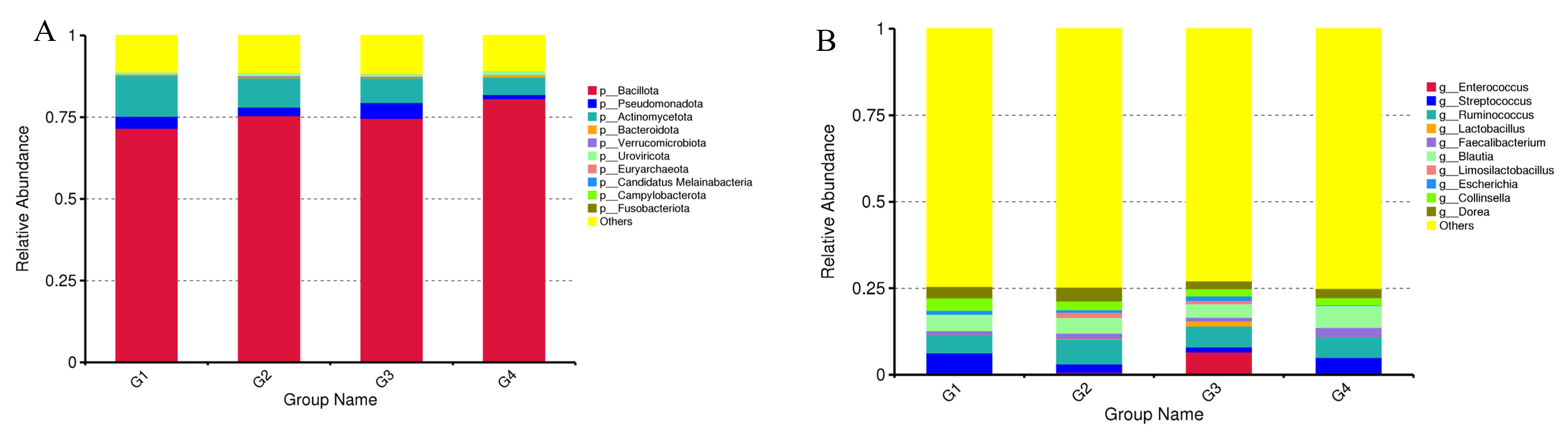

Supplement: Supplementary Figure 1 — Taxonomic composition of gut microbiota at phylum and genus level. (A) Relative abundance of dominant bacterial phyla across groups G1–G4. (B) Relative abundance of dominant bacterial genera across groups G1–G4. Taxa with low relative abundance were grouped as “Others”. [file Image1.tif]
